# Robotic-Assisted Knee Arthroplasty: Insights and Implications From Current Literature

**DOI:** 10.7759/cureus.50852

**Published:** 2023-12-20

**Authors:** Zaid Yasen, Hugo Woffenden, Andrew P Robinson

**Affiliations:** 1 Trauma and Orthopedics, Royal Free Hospital, London, GBR; 2 Surgery and Cancer, Imperial College London, London, GBR; 3 Trauma and Orthopedics, Ministry of Defence, Portsmouth, GBR; 4 Trauma and Orthopedics, Lewisham and Greenwich NHS Trust, London, GBR

**Keywords:** total knee arthroplasty technique, unicompartmental knee replacement, cost assessment, robotic knee surgery, orthopaedic surgery

## Abstract

Robotic-assisted knee arthroplasty has emerged as a promising development, aiming to enhance surgical precision and patient outcomes. This literature review examines the clinical efficacy, cost implications, environmental impact, and potential of telesurgery in robotic-assisted total knee arthroplasty (RATKA) and robotic-assisted unicompartmental knee arthroplasty (RAUKA) relative to conventional techniques. A thorough literature search was conducted across medical databases. Clinical and radiological outcomes of RATKA and RAUKA were extracted and analyzed. Direct costs, operating time, surgeon learning curve, environmental implications, and the futuristic concept of telesurgery were also considered. Subjective patient assessments such as WOMAC, Oxford Knee Score, and SF-36, alongside objective measures like HSS score and KSS, were commonly used. Radiological parameters like hip-knee-ankle (HKA) and femorotibial angle provided insights into post-operative alignment. Evidence indicated sporadic high-level design studies, often with limited samples. Cost remains a major constraint with robotic systems, though high-volume cases might offset expenses. Environmental assessments revealed robotic surgeries generate a higher carbon footprint. Telesurgery, an evolving field, could transcend geographical boundaries but is not without challenges, including high costs, latency issues, and cyber threats. While robotic-assisted surgeries may hold promise in the future, substantial barriers, including acquisition costs, potential surgeon deskilling, and environmental concerns, need addressing. Greater robot utilization may drive costs down with more competitors entering the market. Continued research, especially multi-center RCTs, is pivotal to solidifying the role of robotic systems in knee arthroplasty.

## Introduction and background

The field of orthopedics is prominent within the medical world as it is at the forefront of adopting new technologies and innovations to enhance and advance practice. The use of total knee arthroplasty (TKA) as a treatment for osteoarthritis (OA) has been standard practice for approximately 50 years [1]. It continues to be the most common surgical treatment for OA of the knee, with 110,000 TKAs performed in 2017 in the UK alone [2]; however, its satisfaction rate remains variable with figures as low as 75% among patients [3,4].

A more recent advancement is the introduction of unicompartmental knee arthroplasty (UKA) which is gaining popularity as a more cost-effective, shorter operation with reduced complications [5]. In fact, NICE guidelines now advocate offering patients the opportunity to undergo a UKA where they are suitable for the operation [6], although this is not necessarily reflected in practice across the country due to unfamiliarity with the procedure and schools of thought disputing its value over TKA. Thus, attention is directed towards whether the technological application can improve the surgical parameters of established procedures.

The use of robotics is relatively new but not completely novel in the surgical field [7], with its use being well-established in specialties such as urology [8]. Its use in orthopedics is gaining popularity in arthroplasty but remains a dubious topic as to whether it holds any superiority over conventional knee arthroplasty. This literature review will discuss with types of robotic assistance systems used in knee arthroplasty, the current evidence for its use as compared to conventional arthroplasty, the limitations to application, and the future direction.

The role of robotic assistance in knee arthroplasty

The TKR aims to improve a patient's pain and functionality lost through the OA pathology by restoring the joint line to maintain a balance of flexion-extension gaps, optimizing the mechanical axis and Q angle whilst preserving surrounding soft tissue [1]. Common mechanisms of failure in knee arthroplasty include post-operative loosening and instability, which is heavily influenced by the prosthetic alignment. The skill and focus of the surgeon are critical in ensuring correct prosthetic alignment, which opens up the possibility of human error leading to decreased post-operative functionality, return to theatre, and as such increased morbidity and mortality risk [9].

The use of robotic assistance systems aims to reduce the likelihood of human error and deviation from the most optimal anatomical alignment. The robotic assistance systems vary in their level of autonomy as well as the navigation systems used, relying either on pre-operative 3D imaging such as high-resolution CT or intra-operative anatomical mapping [10].

Passive robotic systems such as OMNIBot [11] provide the lowest degree of input and simply orientate the entry position and direction of the surgical apparatus. These have limited use in knee arthroplasty.

Semi-active systems include Mako, VELYS, and Rosa, which still allow the surgeon to be in control of the surgical maneuvers but will assist in remaining within the correct planes and boundaries by providing feedback that may be haptic, auditory, or visual. These types are most commonly used in knee arthroplasty [2,10,12,13].

Fully active robotic assistance is provided by systems such as ROBODOC and Caspar. Anatomical boundaries are determined preoperatively using 3D imaging. Whilst the surgeon makes the initial approach and protects the soft tissues, the robot executes the physical resections of the femoral and tibial components [2,10].

Common clinical parameters for assessing knee arthroplasty

Subjective Measures

The Western Ontario and Universities Arthritis Index (WOMAC), Oxford Knee Score, and the Short-Form 36 (SF-36) are self-assessment questionnaires completed by the patient which subjectively assess multiple parameters of patient pain, stiffness, and ability to perform activities of daily living [14,15].

Objective Measures

The Hospital of Special Surgery (HSS) score and the Knee Society Score (KSS) are dual rating systems consisting of patient self-assessment parameters of functional ability as well as a clinician-led assessment of the knee joint itself, thus decreasing the risk of reduced scores confounded by patient’s frailty due to increasing age between pre- and post-operative time periods, as well as providing an objective measure by a trained clinician [15,16].

Common radiological parameters to assess knee arthroplasty

The hip-knee-ankle (HKA) assesses the angulation between the mechanical axis of the femur and tibia, allowing assessment of valgus and varus deformity as well as post-knee arthroplasty alignment. It is measured via long-leg x-rays whilst the patient is standing. The Femorotibial angle measures the anatomical axis between the articulating surfaces of the femur and tibial plateau in the coronal and sagittal planes. Femoral and tibial slopes are also commonly measured [2,10,17].

## Review

Medical databases including PubMed, Scopus, Web of Science, and Embase were used with the keywords “robot,” “conventional,” “total knee,” “unicompartmental knee,” “systematic review,” “randomized control trial” in the title and abstract searched.

Comparing outcomes in conventional TKA (CTKA) and robot-assisted TKA (RATKA)

There is a growing body of research comparing CTKA and RATKA. A 2020 systematic review by Agarwal et al. [2] reviewed the clinical and radiological outcomes of CTKA vs RATKA. Twenty-two papers were included in the study. The studies varied between semi-active (Mako) and active (ROBODOC, Caspar) systems. All papers tested clinical outcomes of which 12 reported significant improvement in the RATKA group, while 14 of the 16 papers that assessed radiological outcomes found superiority in the RATKA group. Only two of the papers included were randomized control trials (RCTs) both of which were performed with the fully active ROBODOC.

A meta-analysis of the same data showed, on the whole, statistically significant superiority in functional outcomes in the RATKA group, with significant improvement in HSS and WOMAC scores whilst KSS had no significant difference. Regarding radiological outcomes, CTKA was found to have a significantly increased risk of radiological outliers in the mechanical axis, where a 3° deviation from neutral in the coronal plane was the parameter boundary. Of course, given that the functional scores all include subjective measures, the low number of RCTs taken into account should be noted [2]. Surveys of public opinions of robotics in orthopedics show patients expect fewer complications and less pain than conventional methods, and half the answering population stated they would prefer a less experienced surgeon using a robot to operate on them and a high-volume surgeon using conventional methods to do so [18].

A further systematic review in 2022 by Mullijali et al. whose database differed from Agarwel revealed less promising results. Over the 13 studies, three were RCTs and a total of 2,112 knee arthroplasties were included in the systematic review. Of the nine studies comparing functional outcomes, only two found any significant improvement in the RATKA group with subjective and objective measures being used. One of the six papers assessing radiological outcomes found improvement, and the six papers comparing complication rates showed no difference. Of the 13 papers, six reported longer operative time with the RATKA group [9].

Direct comparison of conventional UKA (CUKA) and robot-assisted UKA (RAUKA)

The availability of high-quality study designs comparing CUKA to RAUKA is similarly sparse. A 2021 systematic review and meta-analysis analyzed 16 studies assessed over 50,000 UKAs; however, over 35,000 came from one large retrospective study. The study determined a lower complication and revision rate in the RAUKA group, however, there were numerous drawbacks to the meta-analysis, with only 0.79% of patients being drawn from RCTs. Furthermore, the maximum follow-up period was five years, with some studies not looking past 10 months [19].

Further considerations

The results show a weak spread of data with sporadic studies with high-level evidence design, usually comprising small sample sizes. Moreover, the presence of long-term data is lacking in both RATKA and RAUKA. For widespread adoption of a technology, the advantages must be shown to outweigh the connotations.

Cost

Cost is always a major determinant in the adaptation of new technologies, especially in systems such as the National Health Service (NHS) where resource allocation is always an issue. The start-up costs of attaining the machinery are vast, with Stryker reportedly quoting the Mako robot as $1.25 million with a further annual cost of $100,000 and $1,000 worth of equipment per case for hip arthroplasty [20]. Its cost-effectiveness is therefore heavily determined by the volume of cases. A US study employing the Markov model to assess cost-effectiveness of RATKA found that based on an acquisition cost of $706,250 for the system and equipment, a total of 42 annual RATKA would make the system cost-effective based on a $50,000/quality-adjusted life year (QALY) willingness to pay (WTP) [21]. It should be noted, however, that whilst the NHS states a WTP of £20,000-30,000/QALY for TKA, the actual cost of a CTKA procedure including the five-year follow-up is £7458 [22], meaning the actual number of RATKA procedures that would need to be conducted would be much higher.

Where high-volume RATKA is performed, the costs appear to be recuperated, as shown 2022 retrospective cost-analysis study in a center that exclusively performed RATKA since the robot acquisition in 2016 [23]. It did report a significantly reduced 90-day episode-of-care cost for RATKA compared to CTKA due to the reported reduced post-operative analgesia used, length of stay, and community support requirement. This was however a single-centre, single-surgeon study.

Operative Duration

RATKA is associated with a longer operative duration which typically corresponds to a higher intra-operative cost, as well as a greater risk of anesthetic complications, venous thromboembolism, and surgical-site infections [24]. Despite this, however, comparisons of complications between RATKA and CTKA have not reported any statistical significance as mentioned previously.

Learning Curve

Further intra-operative costs and extensions to operative time will be additionally incurred by the learning curve of using active systems with studies finding between seven and 43 cases being required before operative time reached a baseline and confidence in theatre was achieved. Notably, the same studies found that the accuracy of implant positioning did not differ between the first case and subsequent cases [25-27].

Risk of Surgeon Deskilling

Given the level of input provided by fully active systems that execute the surgical maneuvers, it is reasonable to raise the concern that this may lead to a loss of skills for the surgical workforce, especially in cases where high-volume cases are being performed. Whilst the outcomes of RATKA are comparable to CTKA, where a robot becomes no longer available due to demand or breakdown, or if the surgeon starts working in an area with no robot assistance system problems will occur with potential risk to the patient. This deficiency in knowledge could then transcend to the subsequent generation of surgeons who rely on seniors for the majority of their skills training.

Computer-Assisted Navigation (CAN)

Whilst not detailed in this report, it should be noted that CAN represents an alternative to robotics as a measure to improve accuracy and functional outcomes. A 2018 retrospective study comparing the two found RATKA to have a shorter navigation time, but overall tourniquet time is the same. Further research is needed to evaluate the relative advantages [28].

Environmental Impact

Whilst there is a lack of research evaluating the carbon footprint of a RATKA or RAUKA, we must be consistently mindful of the long-term impacts when advocating widespread usage of a new technology. Compared to laparoscopic surgery, which already utilizes more waste than traditional open surgery, robotic-assisted surgery was found to result in 43.5% higher greenhouse gas emissions with a 24% increase in waste produced [29].

Telesurgery

The use of robotics has the potential to bring forward a revolutionary era of telesurgery, which could help to bridge gaps in surgical service determined by a patient’s geographic location, especially in low and middle-income countries (LMIC). It would help circumvent the financial and logistic issues of travel for patients and surgeons, especially given the recent COVID-19 pandemic, as well as allow leading experts across the country of different specialties to liaise together.

The drawbacks are significant, however; for example, time latency is an issue that can represent a safety concern, especially in poor network areas. 5G networks can reduce the latency to 0.01s, however, are estimated to cost approximately $100,000 annually. There is even the theoretical risk of cyber security threats to take into account, with its required safety measuring incurring additional costs [30].

## Conclusions

The use of robotics in TKA remains a topic that has surgeons on either side of the fence. The evidence body to show its superiority over CTKA is still currently weak, and due to the currently understandable lack of widespread adoption, the availability of high-quality trials is very slow to grow in number. Worries remain regarding the deskilling of surgeons, while the acquisition and maintenance costs represent a massive barrier. However, a greater uptake in the usage of robots will decrease the price per case as well as decrease the fixed costs for the production of robots, along with potentially reducing the market price as more competitors come into play in response to demand. While it is true that the current evidence does not overwhelmingly point to its use, this is not an indication to stop our exploration of its benefit. Innovations do not always present an immediate advantage - the concept of the internet was thought to be a fad when it first arrived. Multi-center RCTs should be performed with regular systematic reviews to assess the current state of evidence, comparing CTKA with RATKA and existing CAN, as well as comparing different robot types against one another.
